# Atrial functional mitral regurgitation: definition, features, and unknowns

**DOI:** 10.1007/s00392-025-02696-3

**Published:** 2025-06-23

**Authors:** Frank A. Flachskampf, Ruxandra Beyer, Wolfgang Fehske

**Affiliations:** 1 Department of Medical Sciences, Uppsala University, 751 85 Uppsala, Sweden; 2 Clinical Physiology and Cardiology, Uppsala University Hospital, 751 85 Uppsala, Sweden; 3 Heart Institute Cluj-Napoca, University of Medicine and Pharmacy Cluj-Napoca, Cluj-Napoca, Romania; 4 Praxis Rheingalerie, Hauptstraße 39-41, 50996 Cologne, Germany

**Keywords:** Mitral regurgitation, Echocardiography, Cardiac imaging, Valvular heart disease, Left atrium

## Abstract

Atrial functional mitral regurgitation (AFMR) is a form of functional mitral regurgitation as yet insufficiently recognized and characterized. The driving cause is atrial, not ventricular dilatation, usually due to long-standing atrial fibrillation, and often in association with left ventricular diastolic dysfunction and heart failure with preserved ejection fraction. Increase in mitral annular area leads to loss of central coaptation of the leaflets. Because of the normal sized and normally or nearly normally contracting left ventricle, the amount of regurgitation is typically not more than moderate or moderate-to-severe by standard criteria. The time course of AFMR and the relation to symptoms have yet to be elucidated more clearly. There is a large overlap of symptoms and signs between AFMR and heart failure with preserved ejection fraction, making it likely that the role of AFMR in patients with heart failure has been underestimated. This review presents current concepts and published insights into this form of mitral regurgitation.

**Graphical abstract:**

**Figure:**
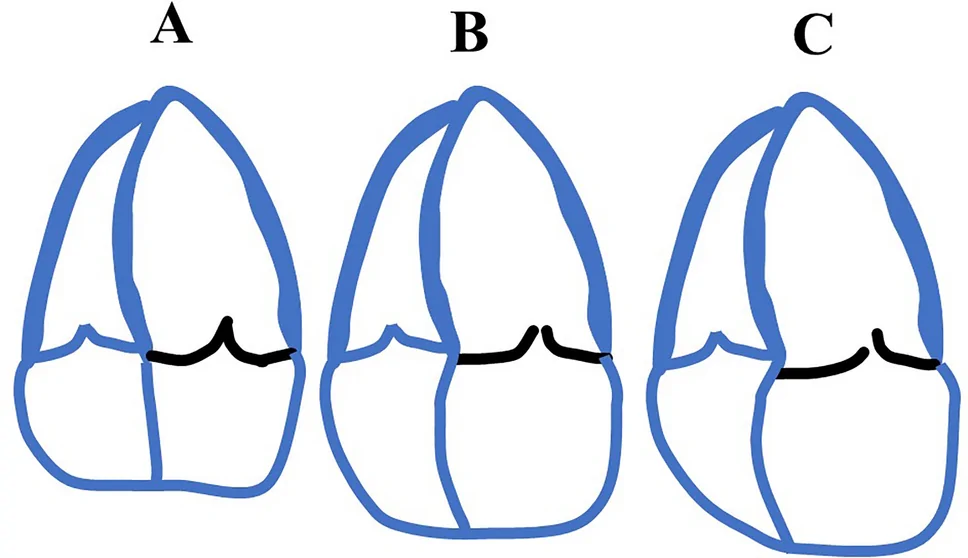

**Supplementary Information:**

The online version contains supplementary material available at https://doi.org/10.1007/s00392-025-02696-3.

## Background

Mitral regurgitation (MR) is traditionally classified as

- Primary, or organic, which may be acute or chronic, typically due to mitral valve prolapse, flail, endocarditis, papillary muscle rupture, cleft, or other diseases causing a structural abnormality of the leaflets or subvalvular apparatus; or
- Secondary, or functional, due to pathologic changes originating in non-valvular structures, most often eccentric remodeling of the left ventricle (LV) leading to changed mechanics of the subvalvular and valvular mitral apparatus. The eccentric pull of the papillary muscles of a dilated ventricle on the mitral leaflets leads to a systolic “tenting” of the mitral valve, and to dilatation of the mitral annulus.

Another form of functional MR is atrial functional mitral regurgitation (AFMR), where the causative mechanism is atrial dilatation, typically due to longstanding atrial fibrillation or atrial cardiomyopathy. This form of MR is less common than “ventricular” functional MR (VFMR) [1, 2]. Clearly, there are overlaps between the atrial and ventricular forms of functional MR, and probably both can initiate vicious circles leading to mixed forms of functional MR. However, AFMR occurs without VFMR and has received less attention and has been less clearly characterized than ventricular FMR. In the following, we will briefly review the existing concepts and published data of AFMR.

### Definition and mechanism

**Fig. 1:**
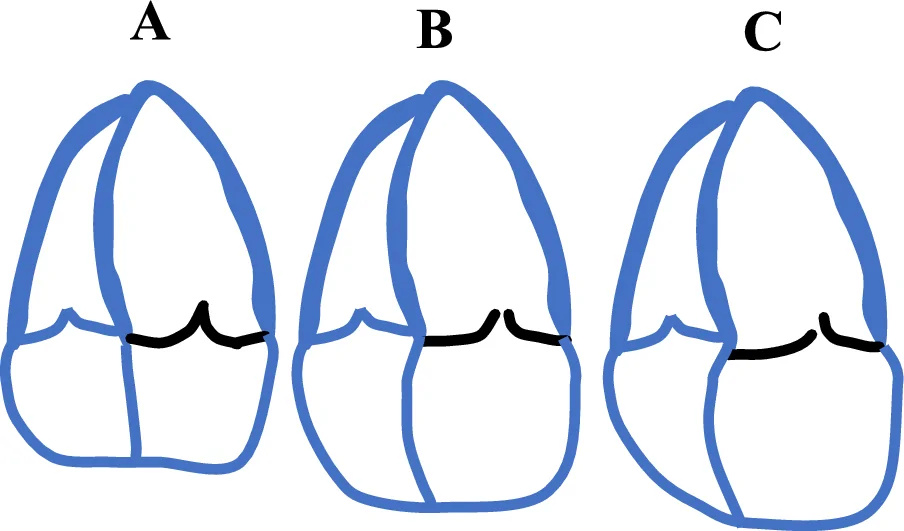
Graphical abstract. Schematic drawing of hypothetic evolution of atrial functional mitral regurgitation with progressive dilatation of the left atrium. **A** Normal anatomy in systole, no mitral regurgitation. Note slight concavity of mitral leaflet bodies towards left ventricle, but no prolapse. **B** Dilated left atrium with a central gap between leaflets; loss of concavity of leaflets towards left ventricle. There is functional atrial mitral regurgitation through the central gap due to disparity between leaflet size and annular orifice size. **C** Further enlargement of left atrium, there is now an “anterior leaflet override” configuration of the mitral valve with tethering of the posterior leaflet and a systolic anterior leaflet tip position on the atrial side of the posterior leaflet, with further increase in mitral regurgitation

AFMR occurs due to atrial dilatation, in the overwhelming majority of cases due to long-standing atrial fibrillation, flutter, or atrial tachycardia, and the development of atrial cardiomyopathy [3]. In this regard, AFMR is similar to functional tricuspid regurgitation. Atrial dilatation leads to mitral annular dilatation, which pulls the mitral leaflets apart and leads to central malcoaptation of the leaflet tips in systole, often in the form of an “override” configuration, or a frank gap between the tips of the leaflets (Fig. 1). Mitral annular dilatation further leads to flattening of the apico-basal profile of the annulus and to reduction in the contraction and expansion of annular area over a heart cycle. Quantitative 3D studies of the relation of the mitral orifice area, which is determined by the annulus size, to the mitral leaflet area have shown that in response to annular dilatation compensatory growth of the mitral leaflets occurs, but that this response is variable and those who develop relevant AFMR show a decreased leaflet area to orifice area ratio [4]. Decreased annular contraction during the cardiac cycle contributes to the leakage. Pronounced tenting is not typical for isolated AFMR, since there is no eccentric pull of the papillary muscles. The regurgitant jet usually is central, although posteriorly directed jets may occur (see last paragraph of this section). The left ventricle by definition is not dilated in pure AFMR and shows normal or near-normal ejection fraction [1, 2]. Since AFMR leads to a volume load of the left atrium, it reinforces atrial dilatation in a vicious circle. In its strict sense, AFMR therefore requires

- A substantially dilated LA, mostly, but not always associated with atrial arrhythmias,
- A normal or near-normal sized LV with normal or slightly reduced ejection fraction, and
- A mitral valve without major structural abnormalities like major tenting, prolapse, or leaflet restriction; however, a small amount of tenting or billowing may be present (Table 1). The “seagull” sign (traction of secondary chordae on the anterior mitral leaflet), typical for ventricular functional MR, is absent.

**Table 1:** Defining features of isolated atrial (AFMR) vs ventricular (VFMR) functional mitral regurgitation; adapted from [1]

Features and parameters	AFMR	VFMR
Left atrial size	Very dilated (> 40 mL/m^2^ according to (1), but probably even larger)	Dilated
Left ventricular size	Normal	Dilated
Left ventricular function	Normal or mildly reduced (EF ≥ 40%)	Typically reduced
Mitral leaflet configuration	No or minor tenting, with loss of concavity towards the LV; possible anterior leaflet override and posterior leaflet tethering	Progressive tenting of both leaflets
Mitral annulus diameter	Dilated (according to (1) > 36 mm in mid-diastole in the four-chamber view or > 35 mm in the parasternal long axis view)	Dilated
Prevalence of atrial fibrillation	Very high (> 50%)	Ca. 30%
Mitral regurgitant jet direction	Central, sometimes posterior	Central, sometimes posterior

Note that mixed AFMR and VFMR occurs

**Fig. 2:**
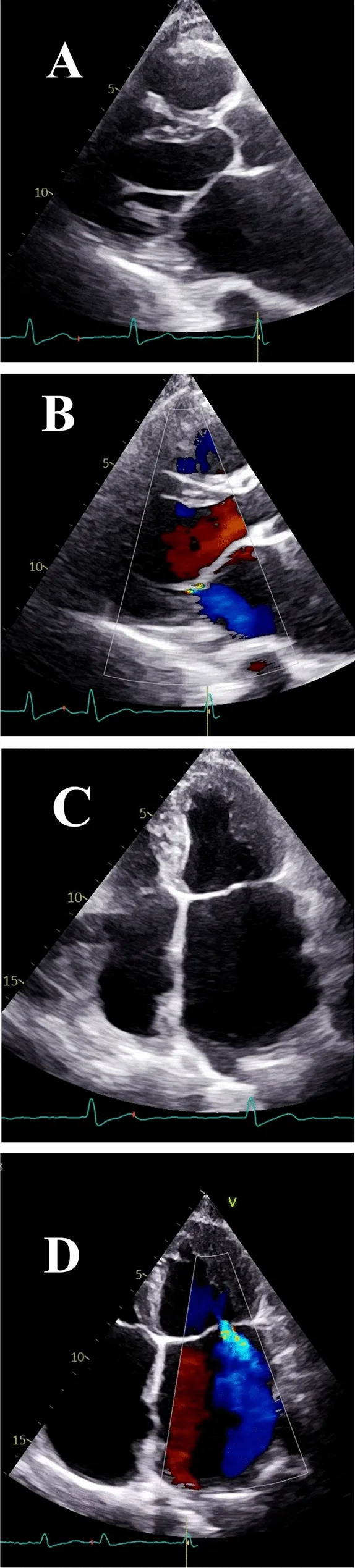
Systolic parasternal long-axis (**A**, **B**) and apical four-chamber views (**C**, **D**) of female 75 year old patient with heart failure symptoms and atrial fibrillation. LV end-diastolic volume 41 mL/m^2^, ejection fraction 65%, LA volume 77 mL/m^2^. LA peak reservoir strain 9%. Moderate AFMR. See also videos 1–3

**Fig. 3:**
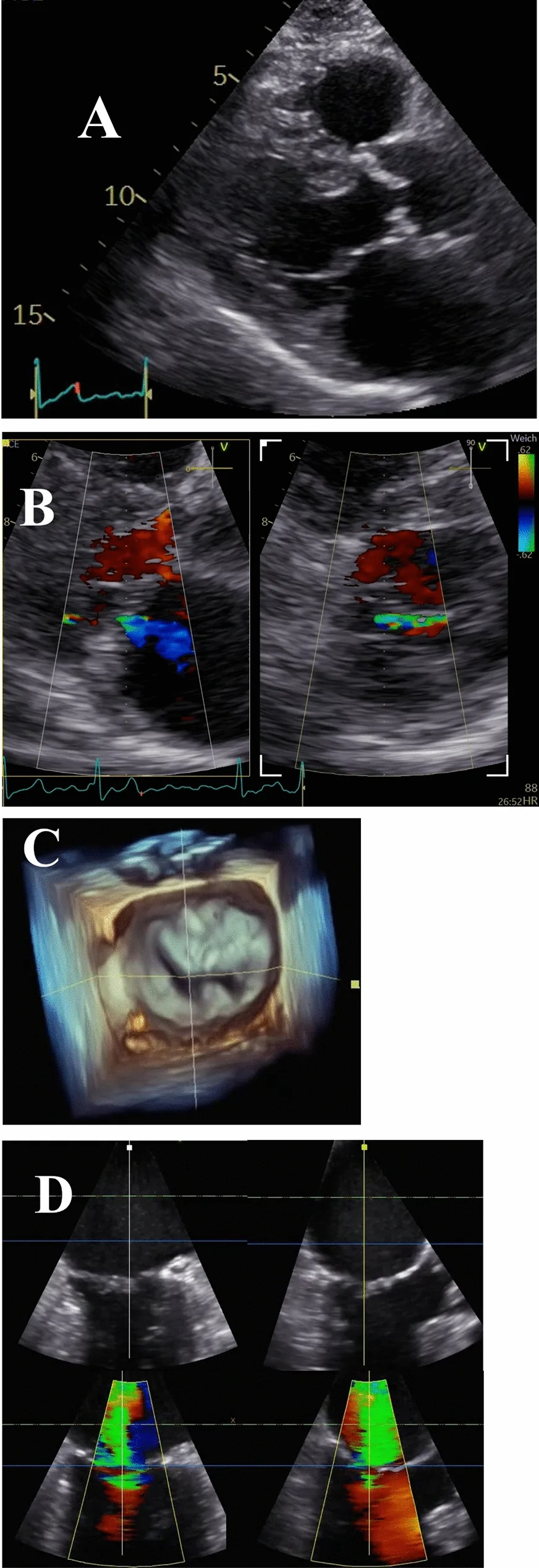
81 years old female patient with permanent atrial fibrillation and mild heart failure symptoms. There is also mild aortic valve disease. LV end-diastolic volume 49 mL/m^2^, ejection fraction 60%, left atrial volume 51 mL/m^2^. **A** Parasternal long axis view showing absence of prolapse/flail or tenting. **B** Biplane parasternal color Doppler view of mitral valve, showing in the short-axis (right) broad jet cross-section filling out entire mitral leaflet closure line. **C** Transesophageal 3D en face view of the mitral valve from the left atrium in systole, showing gap between anterior and posterior leaflet (see also video 4). **D** Biplane transesophageal views of the mitral valve (2D and color Doppler) showing normal leaflets without prolapse or tenting with central mitral regurgitation jet

**Fig. 4:**
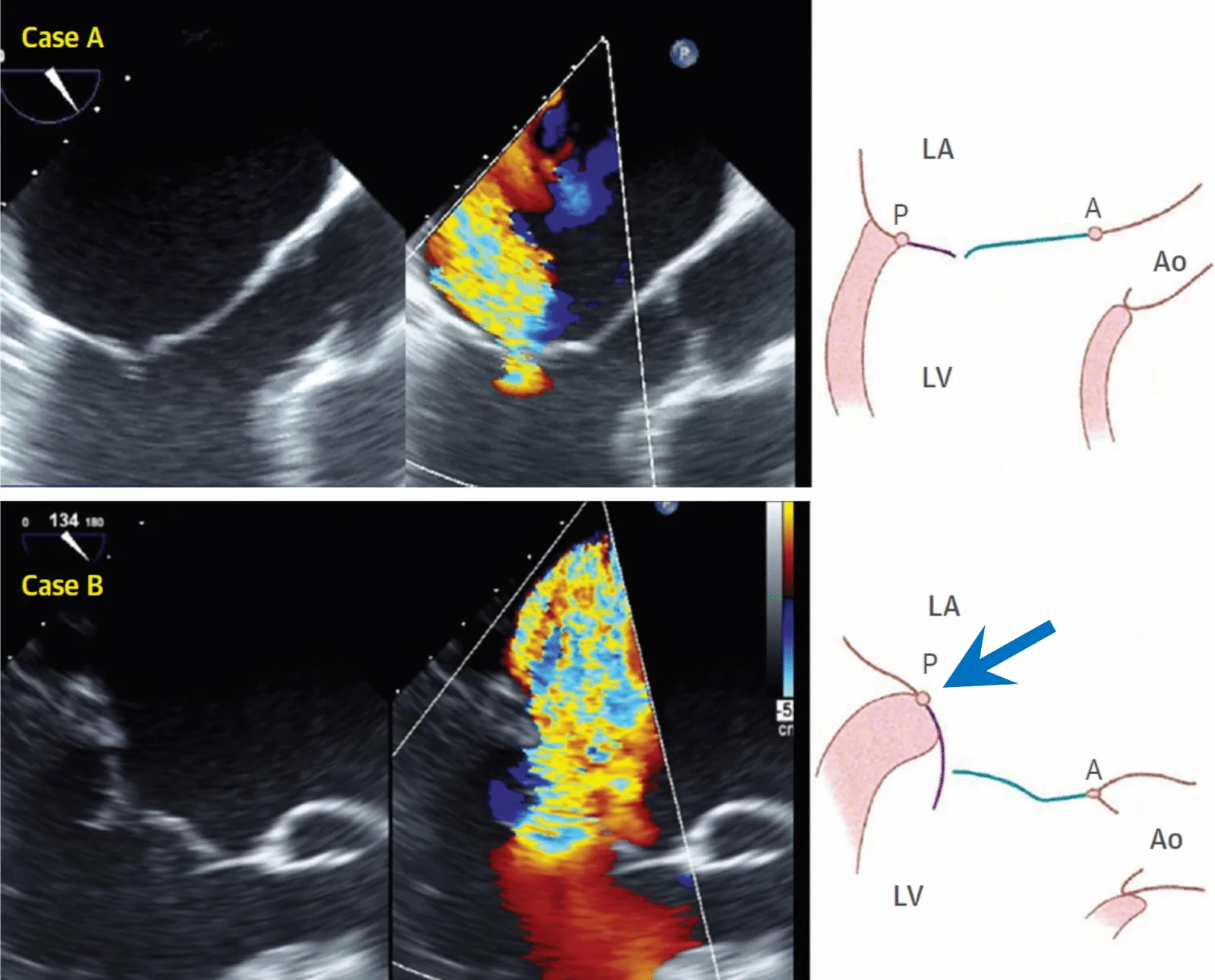
Subtypes of AFMR. In case A, AFMR occurs due to atrial and mitral annulus dilatation with a near-central jet. This is the typical form of AFRM. In case B, atrial dilatation leads to displacement of the insertion of the posterior mitral leaflet (P, arrow) outward on the ridge of the basal ventricular myocardium, producing atriogenic posterior leaflet tethering with a steep posterior leaflet angle. Modified, with permission, from (5)

**Fig. 5:**
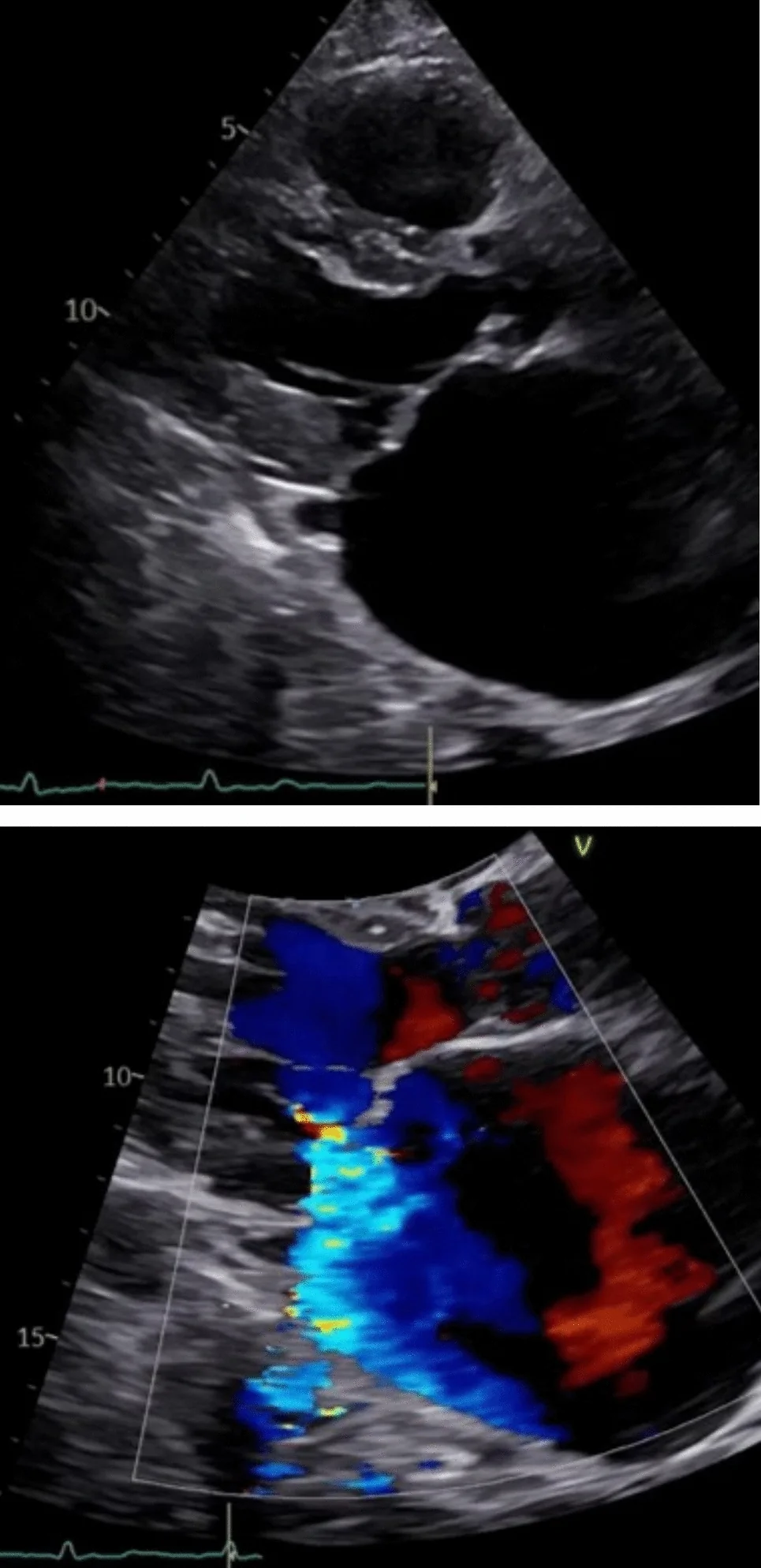
Example of “hamstring” type of AFMR with posteriorly directed eccentric mitral regurgitation jet and massively dilated left atrium. 76 years old female with permanent atrial fibrillation and heart failure symptoms. LV end-diastolic volume 53 mL/m^2^, ejection fraction 68%. Parasternal long-axis view (left 2D, right color Doppler). See also videos 5 and 6

The mechanism of regurgitation is a central leakage (Figs. 2, 3), often caused by an “override” configuration of the leaflets (see Fig. 1). The resulting jet is mostly central. However, some authors [5–8] have described a subgroup of AFMR as the result of a posteriorly located dilatation of the mitral annulus leading to a “herniation” of the annulus, such that the insertion line of the posterior leaflet is displaced outwardly on the ridge of the basal posterolateral ventricular myocardium (Figs. 4, 5). This leads to a particularly strong tethering of the posterior leaflet, with the consequence of a posteriorly directed regurgitant jet. This type of tethering is well seen by echocardiography, but may be localized and hence not visible in all cross-sections of the mitral valve. Several terms have been used to describe this configuration, such as “hamstringing”, “atriogenic”, or atrial tethering MR.

### Hemodynamic features of AFMR

Consequent to the definition of AFMR, the regurgitant volume is not expected to be very large, because a very large mitral regurgitant volume would lead to critical impairment of systemic stroke volume in the absence of left ventricular dilatation: in a 75-year-old male with a 50% ejection fraction and a normal end-diastolic volume of 80 mL/m^2^, total stroke volume is 40 mL/m^2^. With severe AFMR, a regurgitant volume of ≥ 60 mL would be expected. With a body surface area of 1.9 m^2^, this corresponds to ≥ 32 mL/m^2^, leaving an implausibly low systemic (“forward”) stroke volume of maximally 15 ml (8 mL/m^2^). Hence, as long as the LV is not dilated, AFMR is typically rather moderate than severe. The patients, however, seem to be often symptomatic in spite of only moderate or moderate-to-severe AFMR and there is relatively frequently an increase in right-sided pressures. This may be the consequence of a large and stiff left atrium, and hence it is unclear whether the current quantitative cut-offs of severe regurgitation used for primary and ventricular functional mitral regurgitation (ERO ≥ 0,4 cm^2^, regurgitant volume ≥ 60 mL, regurgitant fraction ≥ 50%; [9]) really are adequate for AFMR.

### Relation of AFMR to heart failure with preserved ejection fraction (HFpEF)

AFMR, like HFpEF, is often associated with atrial fibrillation and both occur by definition with normal or almost normal LV size and function. LA pressure may be elevated due to mitral regurgitation, LV diastolic dysfunction, or both. Increased LA pressure and reduced left ventricular global longitudinal strain lead to impairment of atrial mechanical function with reduced LA reservoir strain and, due to increased afterload, also reduced contraction strain. In the presence of atrial fibrillation, LA reservoir strain is even further reduced in both conditions. Features like left ventricular hypertrophy, impaired left ventricular diastolic function, and impaired global left ventricular strain would suggest primarily a disorder of the ventricular myocardium, and hence, HFpEF, but these features may occur with AFMR as well. Thus, in many cases, it is very difficult or impossible to tease out the relative contributions of diastolic dysfunction and of mitral regurgitation to heart failure symptoms, natriuretic peptide elevation, pulmonary congestion, and right sided pressure increase (Table 2).

**Table 2:** Echocardiographic and clinical similarities and differences between AFMR and HFpEF

	AFMR	HFpEF
Heart failure symptoms	Present	Present
Left ventricular size and systolic function	Normal	Normal
Atrial fibrillation	Very frequent	Frequent
Mitral regurgitation grade	Mild to moderate	From absent to moderate
Left atrial function (Reservoir strain)	Decreased	Decreased
maximal E wave velocity	Increased	Increased
E/e’	Increased (high E wave velocity)	Increased (high E, low e’ wave velocity)
Pulmonary pressures	Increased	Increased
Low/absent pulmonary venous S wave	Very frequent	Frequent

A recent study showed that patients with HFpEF and AFMR have more severe heart failure, with lower exercise cardiac output, higher pulmonary pressure, and poorer functional capacity than patients with HFpEF without AFMR [10]. In a retrospective analysis of 515 patients with AFMR [11] collected over nearly 20 years at one center, 38% had a diagnosis of HFpEF and atrial fibrillation, and another 24% had a diagnosis of HFpEF without atrial fibrillation. The patients with HFpEF and atrial fibrillation had larger left atria and worse prognosis than those with HFpEF in sinus rhythm. Another large recent observational study (REVEAL-AFMR, Real-World Observational Study for Investigating the Prevalence and Therapeutic Options for Atrial Functional Mitral Regurgitation; [12]) from 26 Japanese centers included 1007 patients with AFMR. 27% had experienced heart failure hospitalizations, and NTproBNP average (interquartile range) was 1280 (563–2588) pg/mL. Patients in sinus rhythm (20% of 1007) had an average HFA-PEFF score of 5.6, and patients in persistent or permanent atrial fibrillation (66%) and an average HFA-PEFF score of 5 (≥ 5 being considered diagnostic for HFpEF [13]). Hence, a large proportion of AFMR patients present phenotypically as HFpEF and vice versa. These insights may have implications for treatment, indicating that in some HFpEF patients valvular intervention or interventions to re-establish sinus rhythm may be helpful.

### Epidemiology and natural course

In a recent report on the etiology of all cases of moderate or severe MR seen at the Mayo Clinic over 10 years, functional MR made up 65% of a total of 727 MR cases, and about 40% of functional MR cases were diagnosed to be of atrial origin [14]. AFMR increased dramatically with age, being nearly absent under the age of 55 years but the second most frequent etiology ≥ 75 years. Abe et al. [15] showed that in atrial fibrillation patients without other apparent heart disease and a left ventricular ejection fraction ≥ 50%, the incidence of at least moderate AFMR increased from 3% in patients with duration of atrial fibrillation < 1 year to 28% in those with a duration > 10 years. Survival curves in AFMR cases were similar to those in primary MR, with considerable excess mortality compared to expected survival of an age and sex matched general population cohort. It should be pointed out, though, that these were all-cause deaths, not cardiovascular mortality, which presumably implies a different spectrum of causes of death for ventricular vs. atrial FMR.

The natural course of AFMR has received limited attention. A report following a retrospectively defined, relatively low-risk cohort of 635 AFMR patients found that progression to left ventricular dilatation and failure was rare and did not differ between patients with no or mild AFMR and those with more severe regurgitation [16]. In another retrospective cohort of 515 AFMR with a relatively low prevalence (37%) of atrial fibrillation history, left atrial reservoir strain (average: 14.8 ± 7.6%) was associated closer with the outcome of death or heart failure hospitalization than left atrial volume index [11]. The Japanese REVEAL study followed 1007 AFMR patients for a median of 2.9 years and reported 189 all-cause deaths, 141 hospitalizations for heart failure, and 68 cardiovascular deaths [17], with no difference in death or heart failure hospitalization between patients in sinus rhythm or with atrial fibrillation. See Table 3 for selected published data.

**Table 3:** Published demographic and hemodynamic parameters of atrial functional mitral regurgitation

References	Age (years)	Degree of MR	EROA (cm^2^)	LAVi (mL/m^2^)	LVEDVi (mL/m^2^) or mm if LVEDD	Ejection fraction (%)	% persistent afib
Gertz 2011 [20] n = 53, 16 fem	61.6 ± 8.4	Mod/sev		32 ± 13	LVEDD 50 ± 3 mm	62 ± 7	62
Mesi 2021 [8] n = 41, 22 fem	70.4 ± 12.9	Severe	0.41	55	54	60	28
Saito 2018 [22] n = 30, 13 fem	77.6 ± 8.2	Mod/sev		113 + 75	LVEDD 51 ± 8 mm	55 ± 4	
Tamargo 2020 [10] n = 117, 81 female	71 ± 10	78 mild 39 mod No sev		45 ± 15	114 ± 30	62 ± 6	38
Uno 2021 [23] n = 56, 32 fem	77 ± 10	2 + n = 15 3 + n = 18 4 + n = 23	0.30 ± 0.11	83 ± 43	54 ± 18	60 ± 6	100
van Rosendael 2014 [24] n = 49, 15 fem	63 ± 8.7	42 mod/7 sev		60.2 ± 14	51 ± 9	63 ± 6	All afib, 27% persistent
Cramariuc 2023 [11] N = 515, 56% female	71 ± 12	Mod 97%		56 ± 18	63 ± 19	65 ± 7	76%
Naser 2024 [16] n = 635, 51% female	75 (66–81)	Mod/sev or sev 5%	0.18 (0.14–0.23)	49 (44–57)	LVEDD 49 (45.5–53)	62 (58–65)	60%
Kagiyama 2024 [5] and Murata 2025 [25]; REVEAL-AFMR n = 1007, 56% female	78 ± 10	Mod (72%) Mod/sev (16%) Sev (13%)	0.27 ± 0.12	95 ± 64	63 ± 21	62 ± 6	80% afib, 67% persistent/permanent

*BSA* body surface area, *EROA* effective regurgitant orifice by proximal convergence zone method, fem female, *LAVi* left atrial volume indexed for body surface area, *LVEDD* left ventricular end-diastolic diameter, *LVEDVi* left ventricular end-diastolic volume indexed for body surface area, *mod* moderate, *sev* severe, *afib* atrial fibrillation

### Treatment and outcomes

AFMR is amenable to current surgical and interventional mitral repair techniques, apart from mitral valve replacement. In the largest so far published cohort of severe AFMR patients with follow-up, AFMR patients had overall worse survival, more heart failure hospitalizations (and more diastolic dysfunction), and underwent less often valve intervention than similarly aged patients with severe primary MR and normal left ventricular function [8]. Recently, the results of percutaneous edge-to-edge repair in 126 patients with AFMR were published, with good procedural success and symptomatic improvement [18]. Whether this translates into improved prognosis cannot be answered currently. Patients with the “hamstrung” or atriogenic tethering pathology had less success of transcatheter mitral edge-to-edge repair, more residual regurgitation after intervention, and worse prognosis than those with the pure annular dilatation [19].

Since atrial tachyarrhythmias are a key factor in the development of AFMR, ablation of atrial arrhythmias is an appealing treatment option [20]. In a cohort of 136 AFMR patients who underwent atrial fibrillation ablation, MR decreased in 64%, and left atrial volume also decreased [21]. The patients who had sinus rhythm restored had less MR and more reduction in left atrial volume than those who did not. However, the extent of low-voltage areas in the left atrium, thought to reflect left atrial fibrosis or cardiomyopathy, predicted less reduction in MR and left atrial volume, suggesting that in the presence of extensive low-voltage areas AFMR may not be improved by ablation even if sinus rhythm is restored.

### Which aspects of AFMR remain incompletely understood?

The most important open questions related to AFMR are

- The time course of the disease and of left atrial remodeling, and whether LV remodeling occurs in the long term;
- How to define “severe” AFMR given that a normal sized left ventricle precludes very large regurgitant volumes;
- Is a substantial proportion of what we call HFpEF in fact the consequence of AFMR?

## Conclusion

AFMR needs to be recognized and understood as an important type of functional mitral regurgitation with morphological and pathophysiological characteristics that set it apart from other types of MR. It may mimic HFpEF. Similar to a large fraction of functional atrial tricuspid regurgitation cases, the driver of this disease is neither a diseased valve nor the ventricle, but the remodeled atrium, in particular dilatation of the mitral annular area, in close association with the burden of atrial fibrillation over time. This is important not only for diagnosis, but also for the choice of therapy. Avoidance of major left atrial remodeling is crucial for the prevention of AFMR, and the induction of reverse remodeling by rhythm control is the ideal therapeutic principle, if this is not precluded by the presence of extensive atrial cardiomyopathy. The presence of atrial tethering or the “hamstring pathology” is associated with worse interventional results and overall carries a worse prognosis than the “standard” form of AFMR.

## Supplementary Information

Below is the link to the electronic supplementary material.

Supplementary file1 (MP4 1674 KB)

Supplementary file2 (MP4 2528 KB)

Supplementary file3 (MP4 1314 KB)

Supplementary file4 (MP4 1996 KB)

Supplementary file5 (MP4 1320 KB)

Supplementary file6 (MP4 989 KB)
